# Demographic trends of cardiorenal and heart failure deaths in the United States, 2011–2020

**DOI:** 10.1371/journal.pone.0302203

**Published:** 2024-05-29

**Authors:** Joseph J. Shearer, Maryam Hashemian, Robert G. Nelson, Helen C. Looker, Alanna M. Chamberlain, Tiffany M. Powell-Wiley, Eliseo J. Pérez-Stable, Véronique L. Roger

**Affiliations:** 1 Heart Disease Phenomics Laboratory, Epidemiology and Community Health Branch, National Heart, Lung and Blood Institute, National Institutes of Health, Bethesda, Maryland, United States of America; 2 Chronic Kidney Disease Section, Phoenix Epidemiology & Clinical Research Branch, National Institute of Diabetes and Digestive and Kidney Diseases, National Institutes of Health, Phoenix, Arizona, United States of America; 3 Department of Quantitative Health Sciences, Mayo Clinic, Rochester, Minnesota, United States of America; 4 Department of Cardiovascular Medicine, Mayo Clinic, Rochester, Minnesota, United States of America; 5 Social Determinants of Obesity and Cardiovascular Risk Laboratory, Cardiovascular Branch, National Heart, Lung and Blood Institute, National Institutes of Health, Bethesda, Maryland, United States of America; 6 Intramural Research Program, National Institute on Minority Health and Health Disparities, National Institutes of Health, Bethesda, Maryland, United States of America; 7 Minority Health and Health Disparities Population Laboratory, National Heart, Lung and Blood Institute, National Institutes of Health, Bethesda, Maryland, United States of America; University of Calcutta, INDIA

## Abstract

**Background:**

Heart failure (HF) and kidney disease frequently co-occur, increasing mortality risk. The cardiorenal syndrome results from damage to either the heart or kidney impacting the other organ. The epidemiology of cardiorenal syndrome among the general population is incompletely characterized and despite shared risk factors with HF, differences in mortality risk across key demographics have not been well described. Thus, the primary goal of this study was to analyze annual trends in cardiorenal-related mortality, evaluate if these trends differed by age, sex, and race or ethnicity, and describe these trends against a backdrop of HF mortality.

**Methods and findings:**

The Centers for Disease Control and Prevention Wide-ranging ONline Data for Epidemiologic Research database was used to examine cardiorenal- and HF-related mortality in the US between 2011and 2020. International Classification of Diseases, 10 Revision codes were used to classify cardiorenal-related deaths (I13.x) and HF-related deaths (I11.0, I13.0, I13.2, and I50.x), among decedents aged 15 years or older. Decedents were further stratified by age group, sex, race, or ethnicity. Crude and age-adjusted mortality rates (AAMR) per 100,000 persons were calculated. A total of 97,135 cardiorenal-related deaths and 3,453,655 HF-related deaths occurred. Cardiorenal-related mortality (AAMR, 3.26; 95% CI: 3.23–3.28) was significantly lower than HF-related mortality (AAMR, 115.7; 95% CI: 115.6–115.8). The annual percent change (APC) was greater and increased over time for cardiorenal-related mortality (2011–2015: APC, 7.1%; 95% CI: 0.7–13.9%; 2015–2020: APC, 19.7%, 95% CI: 16.3–23.2%), whereas HF-related mortality also increased over that time period, but at a consistently lower rate (2011–2020: APC, 2.4%; 95% CI: 1.7–3.1%). Mortality was highest among older and male decedents for both causes. Cardiorenal-related deaths were more common in non-Hispanic or Latino Blacks compared to Whites, but similar rates were observed for HF-related mortality. A larger proportion of cardiorenal-related deaths, compared to HF-related deaths, listed cardiorenal syndrome as the underlying cause of death (67.0% vs. 1.2%).

**Conclusions:**

HF-related deaths substantially outnumber cardiorenal-related deaths; however, cardiorenal-related deaths are increasing at an alarming rate with the highest burden among non-Hispanic or Latino Blacks. Continued surveillance of cardiorenal-related mortality trends is critical and future studies that contain detailed biomarker and social determinants of health information are needed to identify mechanisms underlying differences in mortality trends.

## Introduction

Heart failure (HF) affects over six million adults and contributes to over 13% of deaths in the US [1]. HF is often associated with several comorbidities, including kidney disease. An estimated 15% or 37 million adults have chronic kidney disease [2]. HF is one of the most common cardiovascular outcomes in patients with chronic kidney disease [3]. Furthermore, cardiovascular-related mortality makes up nearly half of deaths of people with chronic kidney disease [4]. Overall, co-occurrence of HF and kidney disease leads to an increased risk of mortality [3, 5–9] and may play an important role in therapeutic decision making [10]. The public health burden of these diseases is largely unabated and even increasing in younger adults [11, 12].

The interplay between the heart and the kidney is inherently complex [13–17]. In response, attempts have been made to develop and refine a consensus definition for cardiorenal syndrome [18, 19]. Cardiorenal syndrome includes a spectrum of disorders resulting from damage to either the heart or kidney that ultimately impacts the other organ. This occurs through a range of shared mechanisms including inflammation, metabolism, and vascular dysfunction [20]. Despite cardiorenal syndrome and HF sharing many common risk factors, such as hypertension, diabetes, and older age [21, 22], differences in their epidemiology remain poorly characterized and the existing classification is challenging to operationalize in practice, because of the complex pathophysiology of cardiorenal syndrome [19]. As a result, our understanding of how the epidemiology of cardiorenal syndrome may differ from HF across key demographic groups remains limited, especially among populations reflective of the general population and not selected based on disease status.

We decided to start addressing this gap in knowledge by analyzing trends in cardiorenal-related deaths, evaluating if these differed by age, sex, race, or ethnicity, and evaluate how the trends compared against the backdrop of HF mortality in the US from 2011 through 2020.

## Materials and methods

### Mortality assessment and covariate information

We examined US mortality data from 2011 through 2020, using the Centers for Disease Control and Prevention (CDC) Wide-ranging ONline Data for Epidemiologic Research (WONDER) database [23]. At the time of analysis, the Final Multiple Cause of Death Data file available only contained mortality data through 2020. Specifically, we used the Multiple Cause of Death database which provides information recorded on death certificates from US residents from all US counties. Available information included the underlying cause of death and up to 20 additional non-underlying (contributory) causes of death, and demographic information [24]. For clarity, we refer to a “related” cause of death as any mention regardless of being an underlying or contributory cause. IRB approval was unnecessary as CDC WONDER is a publicly available de-identified resource of deceased individuals.

We analyzed available information on decedents with a recorded age of 15 years or older. We used International Classification of Diseases, 10 Revision codes and considered both the underlying cause of death and the contributory causes to identify cardiorenal-related deaths (I13.x) and HF-related deaths (I11.0, I13.0, I13.2, and I50. x). Any decedent with an I13.2 or I13.0 code was classified as both a cardiorenal- and HF-related death. For context, we also captured other kidney-related deaths (E10.2, E11.2, E13.2, I12.0, I12.9, N18.0, N18.1, N18.2, N18.3, N18.4, N18.5, N18.8, N18.9, O10.2, O10.3). Medical certification, usually by a physician, medical examiner, or a coroner, using the standard classification list is needed to determine underlying and contributory causes of death recorded on a death certificate [25]. Race and ethnicity are recorded on death certificates using information collected from an informant, such as the next of kin, or by observation when an informant is unavailable. Decedents were further categorized by age-group (15 to 44, 45 to 64, 65 to 84, and 85+ years), sex (male or female), and race or ethnicity (Hispanic or Latino, non-Hispanic or Latino White [NHW], Black or African American [NHB], American Indian or Alaska Native [NHAIAN], and Asian or Pacific Islander [NHAPI]).

### Statistical analysis

Crude and age-adjusted mortality rates (AAMR) per 100,000 persons were calculated. AAMRs were standardized to the 2000 US standard population [26]. The 2000 US standard is currently considered the default reference population for age standardization and was chosen to improve the comparability with the published literature. Joinpoint Regression Program, Version 4.9.1.0. (National Cancer Institute), was used to identify statistically significant changes in crude or AAMR using the annual percent change (APC) [27]. Joinpoint uses Monte Carlo Permutation methodology that can account for estimated variation at each year at each time point to identify statistically significant changes in temporal trends of mortality. All analyses took place between November 2022 and June 2023.

## Results

**Fig 1:**
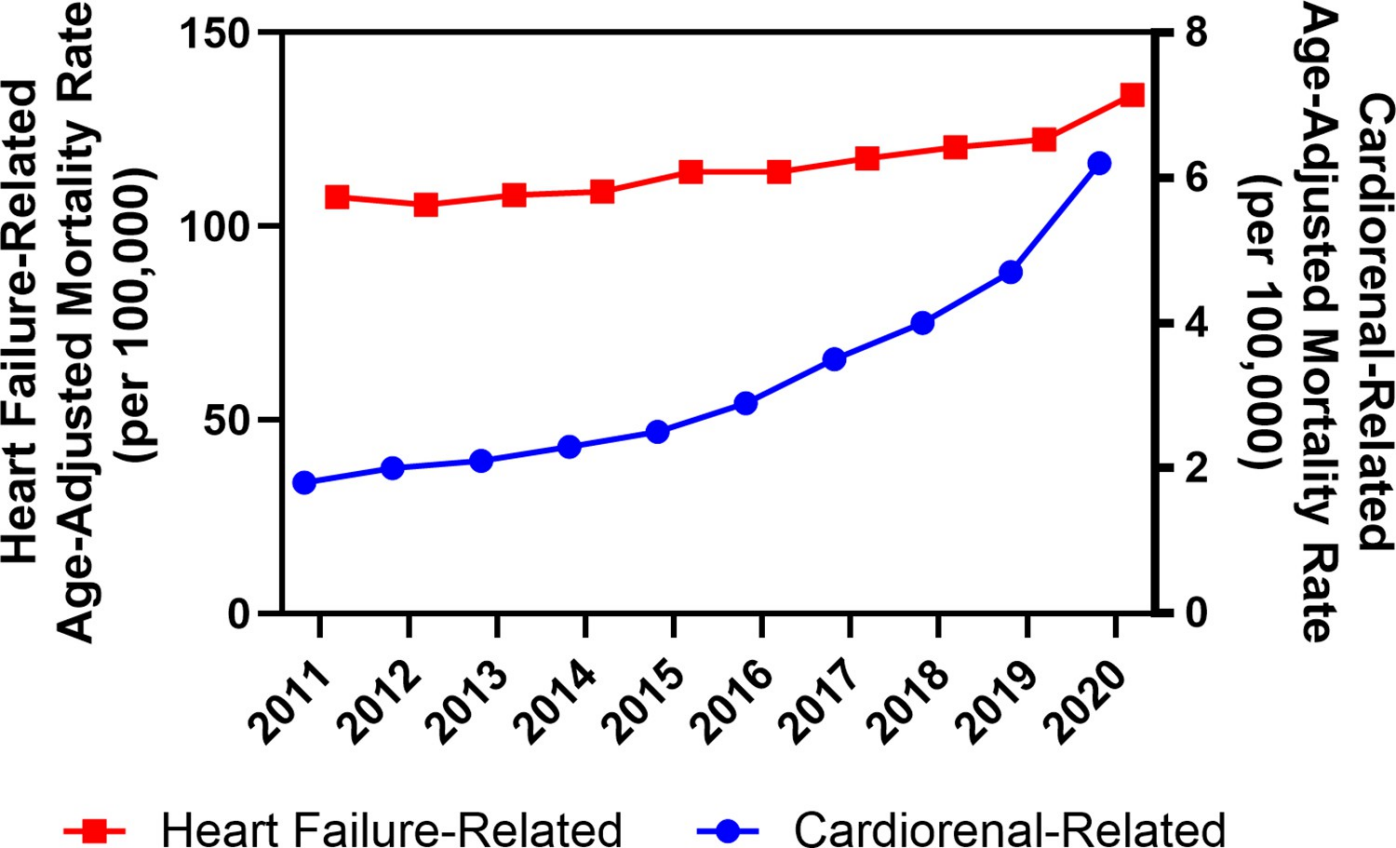
Annual age-adjusted mortality rates, by related cause, 2011–2020. Statistically significant (P < .05) trends in the annual percent change (APC) of age-adjusted mortality rates, identified using Joinpoint Regression analysis, for cardiorenal-related deaths (2011–2015: APC, 7.1%; 95% CI: 0.7–13.9%; 2015–2020: APC, 19.7%, 95% CI: 16.3–23.2%) and heart failure-related deaths (2011–2020: APC, 2.4%; 95% CI: 1.7–3.1%).

Between 2011 and 2020, a total of 97,135 cardiorenal-related deaths and 3,453,655 HF-related deaths occurred among decedents aged 15 years or older in the US (Table 1). Although the largest number of cardiorenal- and HF-related deaths occurred in NHW decedents over the age of 65 years, a greater proportion of cardiorenal-related deaths occurred among decedents who were less than 65 years of age, male, or NHB compared to HF-related deaths. The overall mortality rate for cardiorenal-related deaths (AAMR, 3.25; 95% CI: 3.23–3.28) was significantly lower than HF-related deaths (AAMR, 115.7; 95% CI: 115.6–115.8) (Fig 1). However, the APC was greater and increased over time for cardiorenal-related deaths (2011–2015: APC, 7.1%; 95% CI: 0.7–13.9%; 2015–2020: APC, 19.7%, 95% CI: 16.3–23.2%) compared to HF-related deaths which also increased over that time period, but at a consistently lower rate (2011–2020: APC, 2.4%; 95% CI: 1.7–3.1%).

**Table 1:** Decedent characteristics of cardiorenal and heart failure deaths, 2011–2020.

Group	Population	Cardiorenal-Related		Heart Failure-Related	
	N	N	%	N	%
**Total**	2,606,383,037	97,135	100.0	3,453,655	100.0
**Age Group**					
15–44	1,285,944,033	2,100	2.2	32,985	1.0
45–64	834,939,675	14,253	14.7	317,801	9.2
65–84	422,727,751	39,986	41.2	1,419,128	41.1
85+	62,771,578	40,796	42.0	1,683,741	48.8
**Sex**					
Female	1,334,488,018	48,513	49.9	1,799,157	52.1
Male	1,271,895,019	48,622	50.1	1,654,498	47.9
**Ethnic and Racial Groups**					
Hispanic or Latino	416,255,674	7,515	7.7	179,550	5.2
NHB	326,328,187	19,622	20.2	352,175	10.2
NHW	1,686,507,871	66,092	68.0	2,830,823	82.0
NHAIAN	20,833,439	714	0.7	18,071	0.5
NHAPI	156,457,866	2,866	3.0	65,993	1.9
All Other Groups	N/A	326	0.3	7,043	0.2
**Year**					
2011	250,390,811	4,934	5.1	289,221	8.4
2012	252,769,942	5,443	5.6	291,014	8.4
2013	255,039,716	5,897	6.1	305,396	8.8
2014	257,789,101	6,494	6.7	314,718	9.1
2015	260,402,033	7,326	7.5	337,099	9.8
2016	262,152,444	8,646	8.9	343,922	10.0
2017	264,697,626	10,726	11.0	362,444	10.5
2018	266,281,990	12,567	12.9	379,375	11.0
2019	267,668,677	14,982	15.4	393,249	11.4
2020	269,190,697	20,120	20.7	437,217	12.7

NHW = non-Hispanic or Latino White; NHB = non-Hispanic or Latino Black or African American; NHAIAN = non-Hispanic or Latino American Indian or Alaska Native; NHAPI = non-Hispanic or Latino Asian or Pacific Island

Cardiorenal- and HF-related mortality rates by select demographics are summarized in Table 2. Crude mortality rates increased sharply with age, with relatively low mortality rates among decedents <65 years of age and peak rates among decedents aged 85 years or older (cardiorenal-related: 65.0; 95% CI: 64.4–65.6 and HF-related: 2682; 95% CI: 2678–2686). The APC differed drastically depending on related-cause, with HF-related deaths increasing the most among younger decedents and cardiorenal-related deaths increasing the most among older decedents (S1 Table).

**Table 2:** Mortality rates of cardiorenal- and heart failure-related deaths, by selected demographic factors, 2011–2020.

Group	Cardiorenal-Related			Heart Failure-Related		
	Mortality Rate	Lower 95% CI	Higher 95% CI	Mortality Rate	Lower 95% CI	Higher 95% CI
**Total** ^a^	3.25	3.23	3.28	115.68	115.56	115.81
**Age Group** ^b^						
15–44	0.16	0.16	0.17	2.57	2.54	2.59
45–64	1.71	1.68	1.74	38.06	37.93	38.20
65–84	9.46	9.37	9.55	335.71	335.15	336.26
85+	64.99	64.36	65.62	2682.33	2678.28	2686.38
**Sex** ^a^						
Female	2.74	2.72	2.77	99.78	99.63	99.93
Male	3.93	3.89	3.96	137.18	136.96	137.39
**Ethnic and Racial Groups** ^a^						
Hispanic or Latino	3.03	2.96	3.10	76.22	75.86	76.58
NHW	2.81	2.78	2.83	119.87	119.73	120.01
NHB	7.01	6.91	7.11	130.15	129.71	130.59
NHAIAN	4.13	3.81	4.44	113.03	111.32	114.74
NHAPI	2.24	2.15	2.32	52.33	51.93	52.74

NHW = non-Hispanic or Latino White; NHB = non-Hispanic or Latino Black or African American; NHAIAN = non-Hispanic or Latino American Indian or Alaska Native; NHAPI = non-Hispanic or Latino Asian or Pacific Island

^a^Age-adjusted mortality rate per 100,000

^b^Crude mortality rate per 100,000

Mortality rates also varied significantly by sex. AAMRs were higher among males compared to females for both contributary causes (rate ratio = 1.4 for both). Although we observed a recent sharp increase in mortality rates among females for cardiorenal-related deaths, this trend was not observed for HF-related deaths (S1 Fig).

Significant differences in mortality rates exist across racial and ethnic groups (Table 2). AAMRs for cardiorenal- and HF-related deaths were highest among NHB compared to all other racial and ethnic groups assessed. For cardiorenal-related deaths, AAMRs differed drastically among NHB (7.01; 95% CI: 6.91–7.11) compared to NHW (2.81; 95% CI: 2.78–2.83). Mortality rates among Hispanics or Latinos, NHAIAN, and NHAPI were similar to NHW, with AAMRs ranging between 2.24 and 4.13. For HF-related deaths, mortality rates were the highest among NHB, NHW, and NHAIAN decedents, with AAMRs ranging between 113.03 and 130.15 compared to Hispanic or Latino’s or NHAPIs, whose mortality rates were 76.22 and 52.33, respectively.

**Fig 2:**
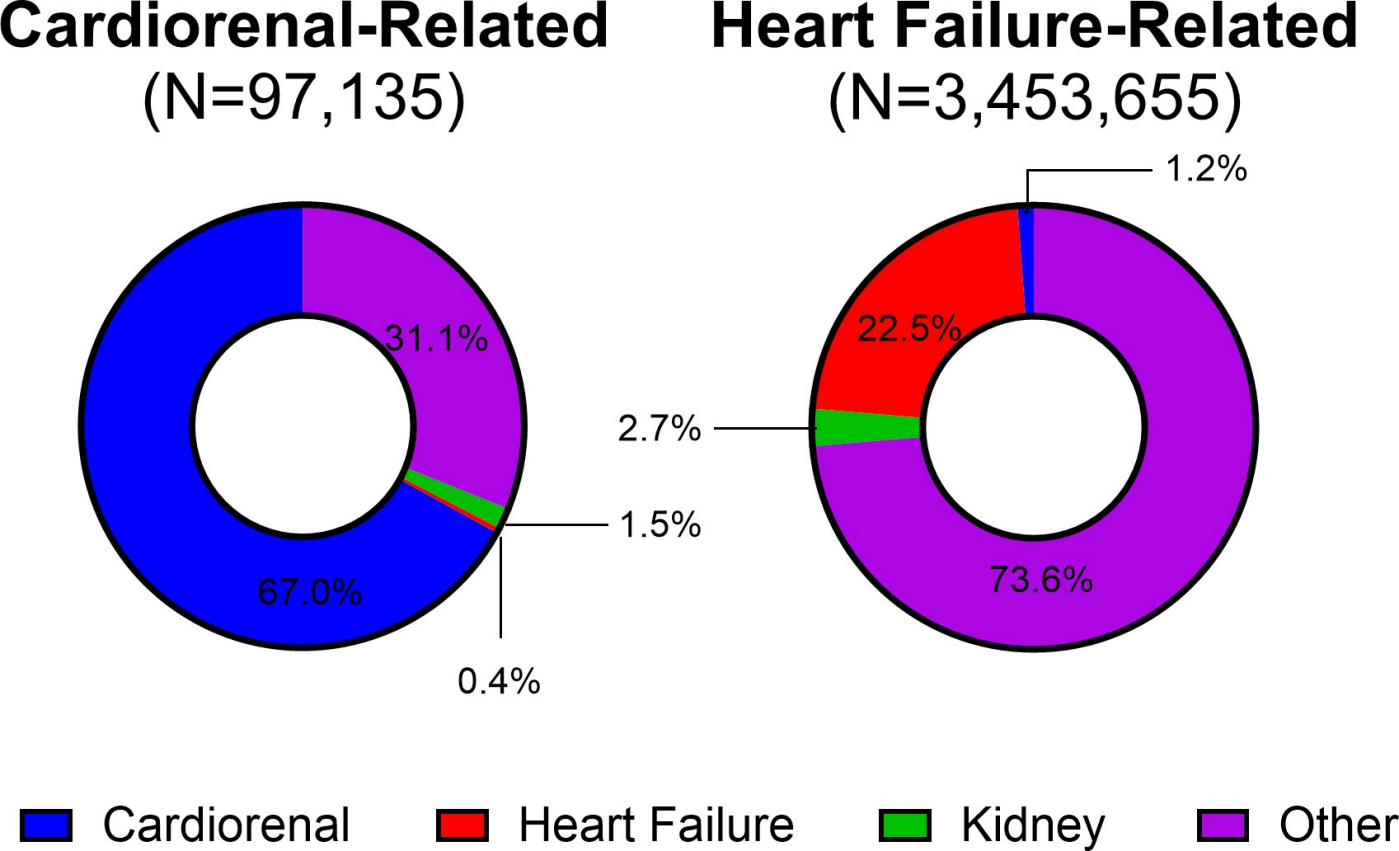
Underlying cause of death among cardiorenal- and heart failure-related deaths, 2011–2020.

**Fig 3:**
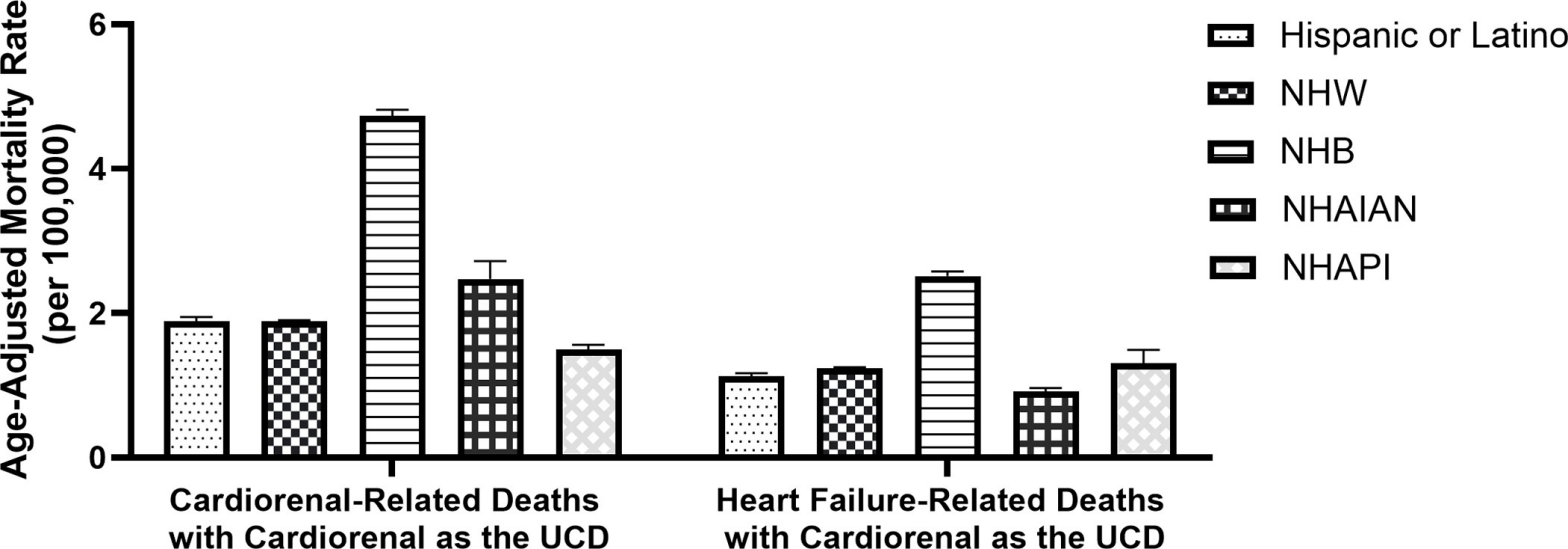
Mortality rates for cardiorenal- or heart failure-related deaths listing cardiorenal as the underlying cause, by racial and ethnic groups. NHW = non-Hispanic or Latino White; NHB = non-Hispanic or Latino Black or African American; NHAIAN = non-Hispanic or Latino American Indian or Alaska Native; NHAPI = non-Hispanic or Latino Asian or Pacific Island; UCD = Underlying Cause of Death.

The underlying cause of death recorded for cardiorenal- and HF-related deaths is summarized in Fig 2. A larger proportion of cardiorenal-related deaths, compared to HF-related deaths, listed cardiorenal syndrome as the underlying cause of death (67.0% vs. 1.2%). Only 22.5% of HF-related deaths listed HF as the underlying cause of death, while 0.4% of cardiorenal-related deaths recorded HF as the underlying cause of death. The proportion of other kidney diseases reported as the underlying cause of death for cardiorenal- was lower than that reported for HF-related deaths (1.5% and 2.7%, respectively). The most common other underlying causes of death were atherosclerotic cardiovascular disease (I25.0), atherosclerotic heart disease (I25.1) and acute myocardial infarction (I21.9) for cardiorenal-related deaths and atherosclerotic heart disease (I25.1), chronic obstructive pulmonary disease (J44.9), and acute myocardial infarction (I21.9) for HF-related deaths. The AAMRs for cardiorenal- and HF-related deaths listing cardiorenal as the underlying cause were greatest among NHB compared to all other racial and ethnic groups assessed (Fig 3).

## Discussion

In this analysis of CDC WONDER mortality data, we found between 2011 and 2020 there were nearly 35 times fewer cardiorenal-related deaths compared to HF-related deaths. Although both cardiorenal- and HF-related mortality rates have increased, the increase in cardiorenal-related deaths was markedly higher than that of HF related deaths. We found overall mortality rates were highest among older male decedents for both related causes but differed across racial and ethnic groups. Notably, NHB had nearly 2.5 times higher cardiorenal-related mortality rates compared to NHW; but similar HF-related mortality rates. Hispanics or Latinos and NHAPI had the lowest HF-related mortality rates of all ethnic and racial groups examined. We also observed that cardiorenal syndrome was rarely listed as the underlying cause of death among HF-related deaths, despite the frequent co-occurrence of HF and kidney disease. Collectively, our results show cardiorenal- and HF-related mortality are highest among older, black, and male decedents with distinct racial and ethnic disparities when comparing cardiorenal- to HF-related mortality, demonstrating the need for continued surveillance of cardiorenal-related mortality.

### Overall trends in mortality

HF-related deaths continuously outnumbered cardiorenal-related deaths between 2011 and 2020. However, our findings align with previous reports suggesting following several years of decline, HF-related mortality rates have begun to level off or even increase slightly in recent years [28, 29]. Unlike HF-related mortality, which increased at a consistently lower rate, cardiorenal-related mortality has sharply increased since 2015. The hypothetical explanations for this observation are likely multifactorial and surely complex. Firstly, increasing awareness of the cardiorenal syndrome could play a role and has been suggested by recent reviews as a key area of emphasis to improve therapeutic management the cardiorenal syndrome [30, 31]. Cardiorenal syndrome is a complex multiorgan syndrome with an evolving history of clinicopathological definitions, for which formal disease classifications have only recently been established [17–19]. Hence, despite the well-known relationship between the heart and kidney and the frequent co-occurrence of disease impacting these organs [3, 5, 6], it is plausible that cardiorenal-related mortality has been underreported until recent years. However, the difference by race and ethnicity suggests that increased awareness is not the primary driver of these temporal trends. Another possible reason for the increased cardiorenal-related mortality rate could be in response to the increasing population burden of kidney disease [32, 33]. The AAMR associated with kidney disease increased by over 50% in the US between 1999 and 2020 [34]. Although, among Medicare beneficiaries with kidney disease, the overall mortality has been decreasing since 2009 [3], which may be related to insured individuals having a higher likelihood of having their disease properly managed rather than a decrease in kidney disease.

### Age and mortality

Cardiorenal- and HF-related mortality rates were highest among the oldest decedents; however, the APC in mortality rates differed in recent years across age groups depending on the related cause. The largest relative increase in HF-related mortality rates were among young adults. This finding aligns with previous reports showing an alarming rise in HF-related mortality among young adults since 2012 [11], in part driven by mortality rates increasing among men, NHB and rural young adult decedents. One plausible explanation, provided by the authors of that study, for the overall increase in HF-related mortality among young adults was the increase in cardiometabolic risk factors and HF incidence among young adults, shown in a recent pooled population-based cohort study [35]. Another was lack of adequate health insurance coverage. This idea is supported by recent evidence from the National Center for Health Statistics showing that males and NHB have lower levels of health insurance coverage compared to other sociodemographic groups [36], however, the same report showed Hispanics were nearly twice as likely to be uninsured as NHB with lower HF-related mortality rates observed in our study. It is likely several mechanisms beyond health insurance may contribute to the observed disparities [37].

Conversely, the largest increases in cardiorenal-related mortality rates were among older adults. One plausible explanation for this is although the overall prevalence of kidney disease remains high [38], the rate among younger adults is relatively low before becoming increasingly common among adults over the age of 65 years [2].

### Sex and mortality

Mortality rates were higher among males compared to females for both related causes across the study period. The finding for HF is consistent with what has been reported for older and younger patients, as well as across racial and ethnic groups [11, 39, 40]. Several sex-specific mechanisms exist that might contribute to the observed elevated risk of mortality observed among males, including higher prevalence of hypertension and differences in disease phenotypes [41]. While mortality rates have been consistently rising for both related causes and sexes, the reason for the sharp increase in mortality rate among females for cardiorenal-related mortality and not HF-related mortality is unclear. From 2019 to 2020, HF-related mortality among males increased by 10% while among females it increased by 8%. We should continue to monitor whether gaps in sex-specific mortality rates widen for HF, as additional years of mortality data are released.

### Race and ethnicity and mortality

We found large, and distinct, racial and ethnic disparities when comparing cardiorenal- to HF-related mortality. NHB decedents had the highest age-adjusted mortality rates for both causes. The persistently higher risk for NHB in HF has been reported across the HF continuum from incidence to hospitalization to mortality [42, 43] and are likely the result of a complex milieu of mechanisms that span health care utilization, social determinants of health, differences in disease treatment, and cardiovascular risk [37].

HF-related mortality rates may depend greatly on whether HF decedents are young or old. For young decedents, NHB are at a much greater risk of HF-related mortality compared to any other racial ethnic group [11]. While in older decedents NHW and NHB both have elevated risk of mortality with NHW at the highest mortality risk compared to other groups [40]. Furthermore, there is some evidence to suggest short-term mortality following hospitalization might be worse for NHW compared to NHB, which may explain why overall rates of HF mortality are elevated in both NHW and NHB compared to other groups [44]. The growing number of younger HF cases and the apparent interaction between age, race, and ethnicity highlights the need to continue monitoring gaps across racial and ethnic groups in HF-related mortality.

While less is known about the racial and ethnic disparities associated with cardiorenal syndrome, we found NHB were at a higher risk of mortality when compared to other ethnic and racial groups. Past reports have consistently shown ethnic and racial disparities for both chronic kidney disease and end stage renal disease, with rates higher among NHB compared to any other ethnic and racial groups [38, 45]. However, it is likely that a similar complex milieu of mechanisms underlies disparities in cardiorenal-related mortality as HF-related mortality. While more work is needed to compare the two, it is reasonable to assume that social determinants of health may be similar among NHB with HF or cardiorenal syndrome, which in turn suggests a larger role for differences in healthcare utilization or disease treatment for the cardiorenal syndrome. This hypothesis is supported by the well-documented history of misuse of race in nephrology which has led to differential treatment and issues measuring kidney function [46]. Thus, it is imperative that future efforts identify factors that differ between the two to better understand the mechanisms underlying the higher risk of cardiorenal-related mortality among NHB not observed in HF-related mortality when compared to NHW.

### Limitations and strengths

Several limitations should be considered when interpreting our results. All causes of death are ascertained using the International Classification of Diseases, 10 Revision codes, which could result in misclassification or variation in the number of contributory causes included on the death certificate and contain limited information on covariates including disease severity. Although the validity of mortality rates by race and ethnicity has been improving, it may vary when reporting beyond Black or White [47]. However, we believe that the benefits of including a more comprehensive approach to describing race and ethnic groups for public health outweigh any perceived limitation in data validity. Lastly, despite the increased risk of HF associated with COVID-19 [48] the impact of infection on these mortality trends was limited due to the last year of data available being 2020; however, a recent publication using data collected from 2019–2021 saw no differences in the 1-year mortality rates in patients with HF [49].

Our study has several important strengths. CDC WONDER uses a standardized data collection approach allowing mortality trends of all death certificates captured in the most recent decade of available data to be compared. Although limited covariate information is available in CDC WONDER, we could assess trends across key demographics including age, sex, race, and ethnicity. Assessing the related causes rather than the underlying cause of death allowed us to capture the collective burden of these cause-specific deaths. Previous studies have shown, in HF, this approach can improve accuracy because the underlying cause of HF may be often misclassified [50].

## Conclusion

The present comprehensive assessment of cardiorenal mortality in the US, across key demographics, directly addresses a critical gap in knowledge in the epidemiology of cardiorenal syndrome and comparing it against a backdrop of HF. We identified that cardiorenal-related deaths were substantially outnumbered by HF-related deaths; however, cardiorenal-related deaths are increasing at an alarming rate with the highest burden among non-Hispanic or Latino Blacks. Continued surveillance of cardiorenal-related mortality trends is critical and future studies with additional covariate information, such as biomarker and social determinants of health, may help identify potential mechanisms underlying differences in mortality trends.

## Supporting information

S1 Table Annual crude mortality rates, by age group, 2011–2020. (DOCX)

S1 Fig Annual age-adjusted mortality rates, by sex, 2011–2020. (PDF)
