# Supplementary material for: Demographic trends of cardiorenal and heart failure deaths in the United States, 2011–2020
Source: PLoS One. 2024 May 29;19(5):e0302203. doi: 10.1371/journal.pone.0302203 (PMC11135744; doi:10.1371/journal.pone.0302203)
Supplement: S1 Table — (DOCX) [file pone.0302203.s001.docx]

**S1 Table. Annual Crude Mortality Rates, by Age Group, 2011-2020.**

|  |  | **Cardiorenal-Related** | | | **Heart Failure-Related** | | |
| --- | --- | --- | --- | --- | --- | --- | --- |
| **Age Group** | **Year** | **Mortality Rate (95% CI)** | **APC (95% CI)** | | **Mortality Rate (95% CI)** | **APC (95% CI)** | |
| 15-44 | 2011 | 0.12 (0.10 to 0.14) | 7.2  (5.3 to 9.2) | | 2.06 (1.98 to 2.14) | 4.4  (3.7 to 5.2) |  |
|  | 2012 | 0.13 (0.11 to 0.15) |  |  | 2.05 (1.97 to 2.13) |  |  |
|  | 2013 | 0.14 (0.12 to 0.16) |  |  | 2.19 (2.11 to 2.28) |  |  |
|  | 2014 | 0.14 (0.12 to 0.16) |  |  | 2.27 (2.19 to 2.36) |  |  |
|  | 2015 | 0.15 (0.13 to 0.18) |  |  | 2.38 (2.29 to 2.46) |  |  |
|  | 2016 | 0.14 (0.12 to 0.16) |  |  | 2.45 (2.37 to 2.54) |  |  |
|  | 2017 | 0.17 (0.15 to 0.20) |  |  | 2.59 (2.50 to 2.67) |  |  |
|  | 2018 | 0.20 (0.17 to 0.22) |  |  | 2.88 (2.78 to 2.97) |  | 16.6  (11.7 to 21.8) |
|  | 2019 | 0.20 (0.18 to 0.23) |  |  | 2.93 (2.83 to 3.02) |  |  |
|  | 2020 | 0.23 (0.20 to 0.26) |  |  | 3.81 (3.70 to 3.91) |  |  |
| 45-64 | 2011 | 1.0 (0.9 to 1.1) | 12.6  (10.9 to 14.3) | | 29.6 (29.3 to 30.0) | 5.1  (4.7 to 5.6) |  |
|  | 2012 | 1.0 (1.0 to 1.1) |  |  | 30.4 (30.0 to 30.8) |  |  |
|  | 2013 | 1.3 (1.2 to 1.4) |  |  | 32.3 (31.9 to 32.7) |  |  |
|  | 2014 | 1.4 (1.3 to 1.5) |  |  | 33.7 (33.3 to 34.1) |  |  |
|  | 2015 | 1.5 (1.4 to 1.6) |  |  | 36.0 (35.6 to 36.4) |  |  |
|  | 2016 | 1.7 (1.6 to 1.8) |  |  | 37.8 (37.4 to 38.2) |  |  |
|  | 2017 | 1.8 (1.7 to 1.9) |  |  | 39.6 (39.1 to 40.0) |  |  |
|  | 2018 | 2.1 (2.0 to 2.2) |  |  | 42.5 (42.1 to 43.0) |  | 12.8  (9.7 to 16.1) |
|  | 2019 | 2.3 (2.2 to 2.4) |  |  | 44.6 (44.2 to 45.1) |  |  |
|  | 2020 | 3.0 (2.9 to 3.1) |  |  | 54.0 (53.5 to 54.5) |  |  |
| 65-84 | 2011 | 5.3 (5.0 to 5.5) | 6.4  (-2.5 to 16.2) |  | 325.6 (323.7 to 327.4) | 1.0  (-0.1 to 2.0) |  |
|  | 2012 | 5.8 (5.5 to 6.0) |  |  | 312.3 (310.5 to 314.1) |  |  |
|  | 2013 | 6.0 (5.8 to 6.3) |  |  | 316.0 (314.2 to 317.8) |  |  |
|  | 2014 | 6.4 (6.2 to 6.7) |  |  | 315.3 (313.5 to 317.0) |  |  |
|  | 2015 | 6.9 (6.7 to 7.2) |  | 19.9  (15.3 to 24.7) | 327.6 (325.9 to 329.4) |  |  |
|  | 2016 | 8.3 (8.1 to 8.6) |  |  | 325.9 (324.2 to 327.6) |  |  |
|  | 2017 | 10.0 (9.8 to 10.3) |  |  | 334.9 (333.2 to 336.6) |  |  |
|  | 2018 | 11.4 (11.1 to 11.7) |  |  | 343.3 (341.6 to 345.0) |  | 6.9  (0.2 to 14.1) |
|  | 2019 | 13.1 (12.8 to 13.5) |  |  | 351.1 (349.4 to 352.8) |  |  |
|  | 2020 | 17.8 (17.4 to 18.2) |  |  | 387.3 (385.6 to 389.1) |  |  |
| 85+ | 2011 | 36.5 (34.9 to 38.1) | 5.8  (0.2 to 11.8) |  | 2545.1 (2532.0 to 2558.1) | 1.8  (1.3 to 2.3) | |
|  | 2012 | 38.8 (37.2 to 40.4) |  |  | 2495.0 (2482.2 to 2507.7) |  |  |
|  | 2013 | 38.5 (36.9 to 40.0) |  |  | 2542.2 (2529.5 to 2554.9) |  |  |
|  | 2014 | 42.0 (40.4 to 43.6) |  |  | 2552.2 (2539.6 to 2564.9) |  |  |
|  | 2015 | 47.9 (46.2 to 49.6) |  | 22.9  (19.8 to 26.0) | 2670.0 (2657.2 to 2682.8) |  |  |
|  | 2016 | 53.9 (52.1 to 55.7) |  |  | 2652.1 (2639.5 to 2664.7) |  |  |
|  | 2017 | 70.0 (68.0 to 72.1) |  |  | 2737.4 (2724.7 to 2750.2) |  |  |
|  | 2018 | 81.3 (79.1 to 83.5) |  |  | 2787.6 (2774.8 to 2800.4) |  |  |
|  | 2019 | 99.2 (96.8 to 101.6) |  |  | 2810.9 (2798.1 to 2823.7) |  |  |
|  | 2020 | 130.0 (127.2 to 132.7) |  |  | 2969.9 (2956.8 to 2983.0) |  |  |
