# Supplementary figures and images for: Demographic trends of cardiorenal and heart failure deaths in the United States, 2011–2020
Source: PLoS One. 2024 May 29;19(5):e0302203. doi: 10.1371/journal.pone.0302203 (PMC11135744; doi:10.1371/journal.pone.0302203)

S1 Figure. Annual Age-Adjusted Mortality Rates, by Sex, 2011-2020.

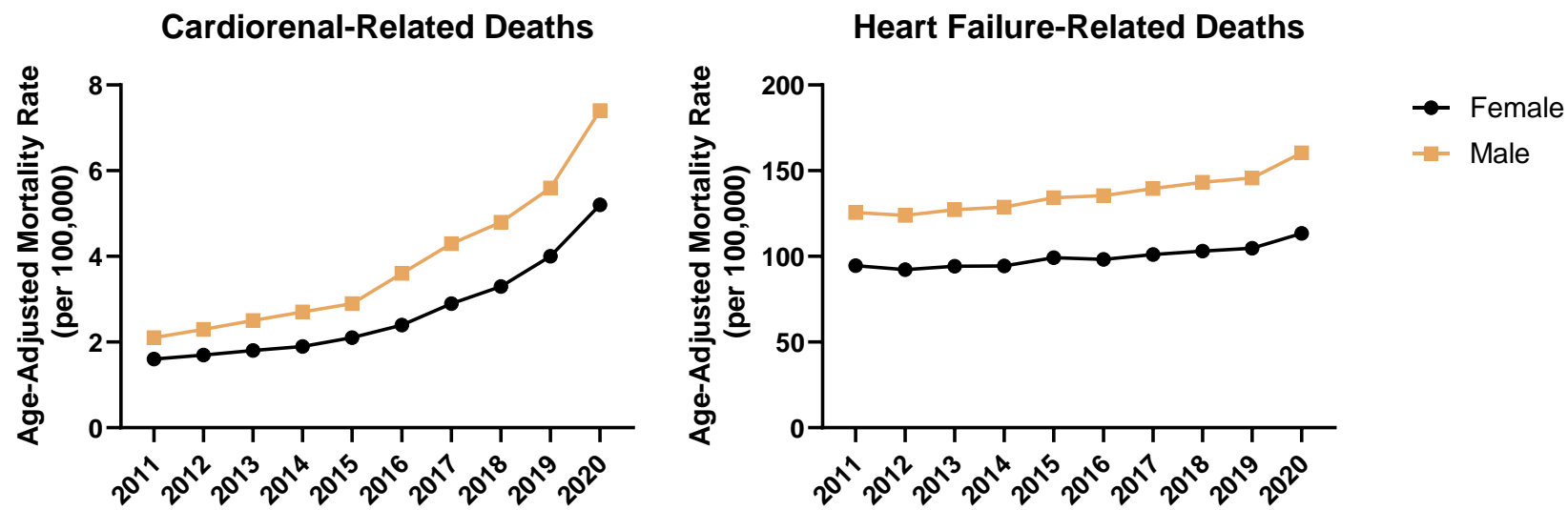

Supplement: S1 Fig — (PDF) [file pone.0302203.s002.pdf]
